# Electrochemically stable tunnel-type α-MnO_2_-based cathode materials for rechargeable aqueous zinc-ion batteries

**DOI:** 10.3389/fchem.2023.1101459

**Published:** 2023-01-24

**Authors:** Yannis De Luna, Asma Alsulaiti, Mohammad I. Ahmad, Hassan Nimir, Nasr Bensalah

**Affiliations:** ^1^ Department of Chemistry and Earth Sciences, College of Arts and Sciences, Qatar University, Doha, Qatar; ^2^ Central Laboratory Unit, Research and graduate studies sector, Qatar University, Doha, Qatar

**Keywords:** energy storage, aqueous rechargeable zinc-ion batteries, manganese oxide, hydrothermal method, electrochemical performance

## Abstract

The purpose of this study is the synthesis of α-MnO_2_-based cathode materials for rechargeable aqueous zinc ion batteries by hydrothermal method using KMnO_4_ and MnSO_4_ as starting materials. The aim is to improve the understanding of Zn^2+^ insertion/de-insertion mechanisms. The as-prepared solid compounds were characterized by spectroscopy and microscopy techniques. X-ray diffraction showed that the hydrothermal reaction forms α-MnO_2_ and Ce^4+^-inserted MnO_2_ structures. Raman spectroscopy confirmed the formation of α-MnO_2_ with hexagonal MnO_2_ and Ce-MnO_2_ structures. Scanning electron microscopy (SEM) confirmed the formation of nanostructured MnO_2_ (nanofibers) and Ce-MnO_2_ (nanorods). The electrochemical performance of MnO_2_ was evaluated using cyclic voltammetry (CV), galvanostatic charge-discharge (GCD) tests in half-cells. CV results showed the reversible insertion/de-insertion of Zn^2+^ ions in MnO_2_ and Ce-MnO_2_. GCD cycling tests of MnO_2_ and Ce-MnO_2_ at 2500 mA/g demonstrated an impressive electrochemical performance, excellent cycling stability throughout 500 cycles, and high rate capability. The excellent electrochemical performance and the good cycling stability of MnO_2_ and Ce-MnO_2_ nanostructures by simple method makes them promising cathode materials for aqueous rechargeable zinc-ion batteries.

## 1 Introduction

Li-ion technology has consistently proven its place as a suitable form of energy storage system with its high energy and power density, among other qualities (Andre et al., 2017). However, LIBs utilize costly materials and are often not environmentally safe, which causes disposal and recovery problems (Costa et al., 2021). Besides these factors, safety concerns surrounding Li-ion batteries (LIBs) have limited their applications, especially in large-scale energy storage systems (Shahjalal et al., 2021). With the constantly growing need for safe, high-performance rechargeable batteries, battery systems beyond Li-ion battery systems need to be considered to uncover any untapped potential. The ideal rechargeable battery requires fast charging, large capacity, and long-term cycling, whilst maintaining safety. Therefore, the shift in research interests towards aqueous rechargeable batteries have grown in the recent years.

Batteries based on multivalent metals, such as aluminium, calcium, magnesium and zinc, could potentially satisfy the demand for large-scale energy storage with low costs, owing to their abundant supply in nature. One of the most promising metal-ion technologies is aqueous rechargeable zinc-ion batteries (ARZIBs) because of the exceptional properties of zinc as anode. For instance, the low material cost, high availability, physical and chemical stability, low toxicity and high safety are ideal characteristics for a battery electrode material (Li and Dai, 2014; Fang et al., 2018; Ming et al., 2019; Tang et al., 2019; Wu et al., 2019; Huang et al., 2020). Until recent developments, zinc-ion batteries have been predominantly applied in primary batteries (non-rechargeable). At the beginning of the last decade, a reversible Zn^2+^ insertion/de-insertion was demonstrated for the first time using tunnel structure of α-MnO_2_ cathode in mild-acid aqueous ZnSO_4_ electrolyte and Zn foil anode (Xu et al., 2012). Besides being a safer alternative to LIBs with low material toxicity, zinc has a high energy density (5855 mA h/cm^3^) (Xu and Wang, 2019) and a moderately high redox potential (Xu et al., 2015). These characteristics improve the suitability of ARZIBs for industrial-scale applications, such as storage systems for renewable energy and electric vehicles (Zhu et al., 2020).

Manganese oxides have gained significant research interests over the last years due to their high working potential, naturally abundant supply, cost effectiveness, and low toxicity (Liu et al., 2021). Specifically, manganese dioxide, MnO_2_, of various crystal structures and Mn oxidation states, has been commonly studied as a cathode for ARZIBs. The various crystal polymorphs such as α-, β-, γ-, and δ-type MnO_2_ that assume different structures (layers, spinels, and tunnels) depending on the positions of MnO_6_ octahedra (Post, 1999; Cheng et al., 2006; Ji et al., 2015; Liu et al., 2018). There are two steps involved in the insertion of Zn^2+^ ions into the structure of the host material (MnO_2_), as shown below:

$${MnO}_{2}+{Zn}^{2+}+2e^{-}\;⇋ZnMnO_{2}$$

$$ZnMnO_{2}+{Zn}^{2+}+2e^{-}⇋2\;{ZnMnO}_{2}$$

According to these reactions, two Zn^2+^ ions can be inserted within one unit of MnO_2_, leading to an overall theoretical specific capacity of 616 mA h/g (308 mA h/g per reaction step). In particular, the structure of α-MnO_2_ consists of tunnels, providing a larger surface area and active sites, which is ideal for the insertion/de-insertion mechanism of Zn^2+^ ions (Alfaruqi et al., 2015).

MnO_2_−based materials have been prepared by different methods, such as through the chemical and electrochemical oxidation of Mn^2+^, the chemical and electrochemical reduction of MnO_4_^−^ or the direct phase transformation from other manganese oxides. It was found that the crystal structure of MnO_2_ affects the cycling stability and electrochemical performance of ARZIBs. Detailed literature reviews (Yan et al., 2012; Kundu et al., 2016; Zhang et al., 2017; Ming et al., 2019; Tang et al., 2019; Wu et al., 2019; Huang et al., 2020) indicate that more studies need to be conducted to understand the mechanisms in which Zn^2+^ ions can be inserted into the host structure at greater depths. In addition, there is a vital need to enhance practical specific capacities to the theoretical value to take advantage of the material’s full potential. Furthermore, present issues that arise during cycling of MnO_2_ materials have been highlighted, such as the dissolution of the host material and the occurrence of irreversible reactions. To overcome such issues, the pre-intercalation of electrode materials with metal ions and water molecules has been explored to stabilize the inner structures (Wang et al., 2019; Zhou et al., 2022).

Herein, a facile hydrothermal method was used to synthesize α-MnO_2_ and pre-inserted Ce-MnO_2_ nanomaterials by redox reaction between Mn^2+^ and MnO_4_^−^ at neutral pH. This research aims to evaluate the cycling performance of MnO_2_-based cathode materials for ARZIBs. The effects of the successful pre-insertion of Ce^4+^ ions into the structure of α-MnO_2_ will be investigated, to determine whether it will facilitate the insertion and de-insertion of Zn^2+^ ions during discharge and charge and improve the capacity and overall performance of the cathode materials. Nanostructured MnO_2_ and Ce-inserted MnO_2_ were synthesized *via* hydrothermal method by reaction of MnO_4_^−^ with Mn^2+^ as manganese sources. The as-prepared nanostructured materials were characterized by different techniques including X-ray diffraction (XRD), Raman spectroscopy, energy dispersive spectroscopy (EDS), scanning electron microscopy (SEM), and transmission electron microscopy (TEM). The electrochemical performance of these nanomaterials as cathode materials for ARZIBs was assessed through electrochemical tests: cyclic voltammetry (CV) and galvanostatic charge/discharge (GCD).

## 2 Experimental

### 2.1 Chemicals used

Potassium permanganate (KMnO_4_) and N-methyl-2-pyrrolidone (NMP) were purchased from Sigma-Aldrich. Manganese sulfate monohydrate (MnSO_4_·H_2_O) was obtained from VWR Chemicals. Cerium(IV) sulfate tetrahydrate (Ce(SO_4_)_2_.4H_2_O) and cerium(III) nitrate hexahydrate (Ce(NO_3_)_3_·6H_2_O) was purchased from Riedel-de Haen. Carbon black was from TIMCAL Graphite & Carbon. Polyvinylidene fluoride (PVDF) was purchased from Arkema. Zinc sulfate heptahydrate (ZnSO_4_.7H_2_O) and zinc foil were purchased from Fisher Scientific Ltd. Filter paper as separator was purchased from Glaswarenfabrik Karl Hecht GmbH & Co. KG, and titanium foil was obtained from Baoji Hanz Metal Material Co.

### 2.2 Methods

#### 2.2.1 Synthesis of MnO_2_-based nanostructures

The MnO_2_-based nanostructures were prepared by hydrothermal method using KMnO_4_ and MnSO_4_ as starting materials. Pure MnO_2_ was synthesized by dissolving a mixture of 3.2 g KMnO_4_ and 5.07 g MnSO_4_.H_2_O in 40 mL deionized water. Ce-inserted MnO_2_ was prepared using the same amounts of KMnO_4_ and MnSO_4_.H_2_O in presence of 1 g Ce(NO_3_)_3_.6H_2_O in 40 mL deionized water. The mixtures were stirred at 50°C–60 °C for 2 h. At the end of the stirring, the pink color characteristic of KMnO_4_ disappeared, indicating the complete reduction of Mn(VII) and the oxidation of Mn(II) into Mn(IV), as shown in the equation below:

$$2\;{{MnO}_{4}}^{-}\left(aq\right)+{3\;Mn}^{2+}\left(aq\right)+{2\;H}_{2}O\to 5\;{MnO}_{2}\left(s\right)+4\;H^{+}\left(aq\right)$$

A dark precipitate of MnO_2_ was formed during the reaction. The suspensions were transferred into separate Teflon jars, then enclosed inside stainless steel autoclaves. The tightly sealed autoclaves were transferred inside an oven and kept at 180°C for 24 h. After the hydrothermal treatment, the content of jars were naturally cooled down to room temperature. The solids were separated by centrifugation at 4000 rpm for 10 min at 20°C, and then washed several times by deionized water until a neutral pH was attained. The washed solids were dried in vacuum oven at 80°C overnight.

#### 2.2.2 Preparation of cathode materials

A slurry containing the active material, conductive material, binder, and solvent was prepared using a ball mill machine (Changsha Tianchuang Powder Technology Co., Ltd. XQM-0.4A), agate grinding Teflon jars and three agate balls (diameter 4 mm). In each jar, 0.75 g of the active material was mixed with 0.15 g of carbon (graphite) used as conductive material, and 0.1 g PVDF used as binder in 3.5 mL N-methyl-2-pyrrolidone (NMP). The agate grinding jar was placed inside the ball miller at 400 rpm for 1 h. The slurry obtained was casted on Ti foil by Doctor Blade and a thick film coater (MTI, MSK-AFA-I), followed by drying in the oven overnight. Then, the dried casted materials were cut into disks using a disk cutting machine (MTI, MSK-T10).

#### 2.2.3 Preparation of Zn/MnO_2_ coin cells

The prepared materials were tested as cathode material in half-cell using Zn metal as counter and reference electrode. The electrolyte used was 3 M ZnSO_4_ aqueous solution. The coin cells (CR 2032) were sealed by a hydraulic crimping machine to finalize its assembly (MTI, MSK-110).

### 2.3 Characterization techniques

Crystal structures were identified by X-ray diffractometry (XRD) (PAnalytical Empyrean X-ray diffractometer, 40 KV/30 mA) at a scan rate of 2°/min between 20° and 90°. Raman spectroscopy (Thermo Scientific™, DXR™ two Raman Microscope) was used to give more details about the structure and morphology of the synthesized solids. Scanning electron microscopy (SEM) analysis using FEI NOVA NANOSEM 450 was conducted to provide information related to morphology of the solids. The same machine was used to identify the elemental composition of solids by energy dispersive X-ray spectroscopy (SEM-EDS). Transmission Electron microscope (FEI, TECNAI G2 TEM, TF20) was used to observe the nanostructured materials and determine inter-planar distances by diffraction patterns.

### 2.4 Electrochemical techniques

Cyclic voltammetry (CV) testing was accomplished using CS350 Potentiostat/Galvanostat (electrochemical workstation). Galvanostatic charge/discharge (GCD) cycling was performed using eight Channel Battery Analyzer (0.005–1 mA, up to 5V) - BST8-WA.

## 3 Results and discussion

### 3.1 Characterization

**FIGURE 1:**
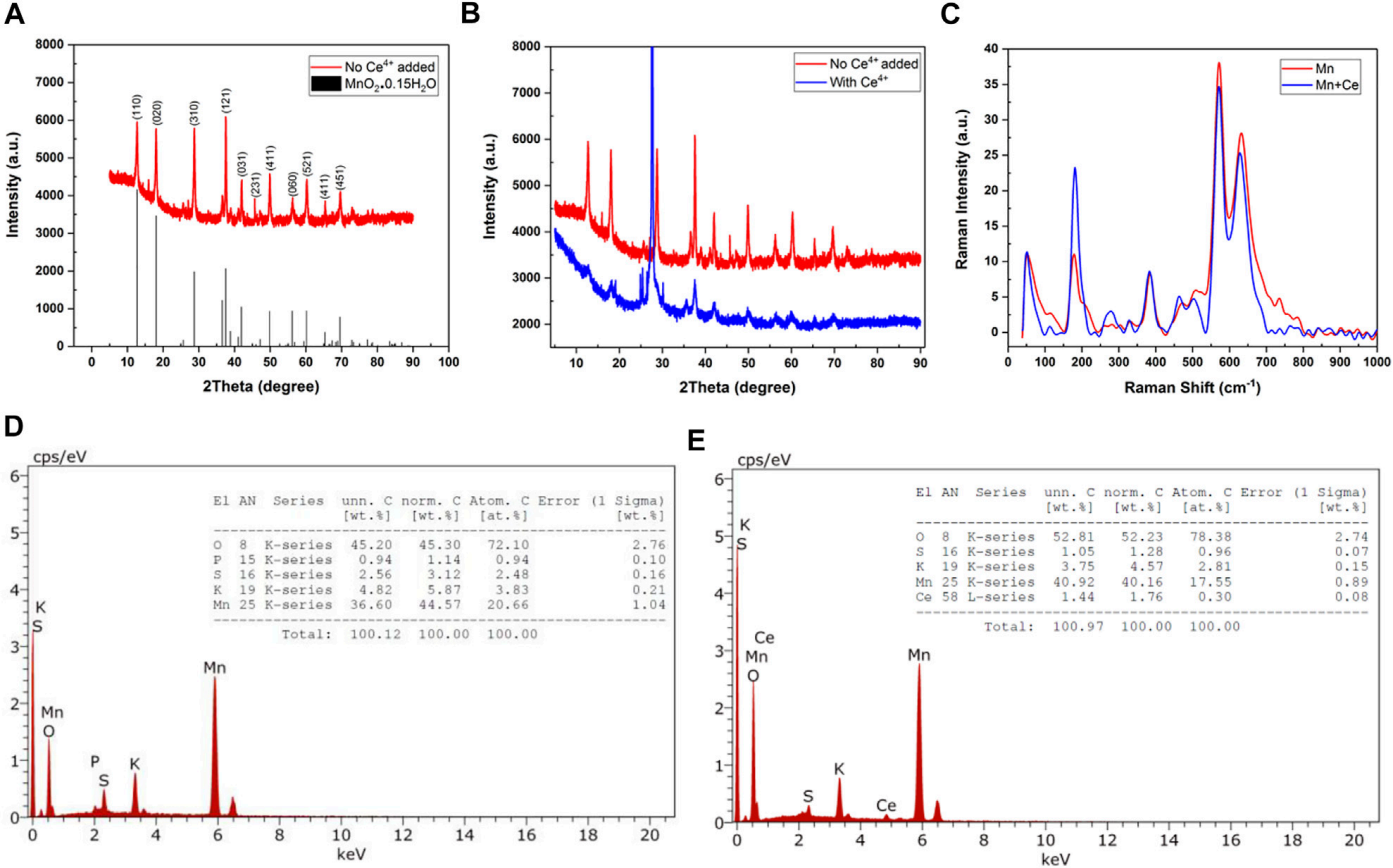
XRD spectra of the solids produced by hydrothermal reaction **(A)** in absence (with α-MnO_2_·0.15 H_2_O as reference) and **(B)** in presence of Ce^4+^ ions. **(C)**Raman spectra of MnO_2_ and Ce-MnO_2_ prepared by hydrothermal method. EDX spectra and elemental analysis results of **(D)** MnO2 and **(E)** Ce-MnO_2_.

The structure of the solids produced by hydrothermal method was analyzed by X‐ray diffraction (XRD) spectroscopy. The XRD spectra of the solid materials produced by the reaction of MnO_4_^−^ and Mn^2+^ in absence and presence of Ce^4+^ are given in Figures 1A, B. The XRD spectrum of the solids produced by the reaction of MnO_4_^−^ and Mn^2+^ in absence of Ce^4+^ match well with the spectrum of α-MnO_2_·0.15 H_2_O. The indexed peaks corresponding to (hkl) planes are shown in Figure 1A. This result demonstrates the formation of hydrated MnO_2_ structure by hydrothermal method. However, the XRD peaks of the solid material produced by the reaction of MnO_4_^−^ and Mn^2+^ in the presence of Ce^4+^ are shifted from those of α-MnO_2_·0.15 H_2_O (see Figure 1B). The observed shifts indicate the pre-insertion of Ce^4+^ into the tunnels of α-MnO_2_ structure (Ce-MnO_2_·0.15 H_2_O).

The average grain size of particles, R, can be calculated from the most intense peak of XRD spectrum using Scherrer formula:

$$R=\frac{K\times \lambda }{\beta \times \cos\theta }$$

Where K is a factor that represents the particle shape with a value equal to 0.9 and the X-ray wavelength is λ = 1.5406 Å. In addition, θ is defined as the diffraction angle for almost intense peak. β is the experimental full width at half maximum of the same peak. The estimated grain sizes were 26.5 nm for MnO_2_ and 400 nm for Ce-MnO_2_, indicating the formation of nanostructures with much smaller nanoparticles in the absence of Ce^4+^ ions.

Raman spectroscopy was performed to identify the structure and morphology of MnO_2_ and Ce-MnO_2_. Raman spectra of MnO_2_ and Ce-MnO_2_ are given in Figure 1C. MnO_2_ and Ce-MnO_2_ present similar Raman shifts located at 182, 390, 570, and 636 cm^−1^. The presence of well-resolved Raman shifts at 570 and 636 cm^−1^ indicates the formation of hexagonal MnO_2_ structure (Hsu et al., 2011). The Raman band at 636 cm^−1^ correspond well with the Raman spectrum of MnO_2_ and can be attributed to the Mn–O symmetric stretching vibrations in MnO_6_ octahedra (Julien et al., 2003; Yin et al., 2015). The Raman band located at 570 cm^−1^ corresponds to the terminal Mn–O stretching in the basal plane of the MnO_6_ sheet in the tunnel-type α-MnO_2_ structure (Hsu et al., 2011). The presence of low-wavenumber Raman bands at 182 and 390 cm^−1^ indicates the insertion of cations into the α-MnO_2_ structure (Yin et al., 2015). The higher intensity of Raman peak located at 182 cm^−1^, in Raman spectrum of Ce-MnO_4_ sample confirms the co-insertion of Ce^4+^ ions, in addition to K^+^ ions.

Energy dispersive X-ray (EDX) analysis was used the elemental analysis at the surface of solid materials. Figures 1D, E present the EDX spectra of MnO_2_ and Ce-MnO_2_ compounds. The EDX spectrum of MnO_2_ shown in Figure 1D confirms the presence of Mn and O, in addition to S, P, and K as impurities. The atomic ratio O/Mn is greater than 2 (O/Mn = 2.65) due to the presence of excess oxygen in SO_4_^2-^ impurities that cannot be removed during washing. The presence of Ce, in addition to Mn and O, was confirmed by EDX, as shown in Figure 1E. Furthermore, impurities of S and K were also detected. The atomic ratio O/Mn was also greater than 2 (3.07) due to the presence of impurities (SO_4_^2−^) and CeO_2_. The atomic ratio of Mn/Ce was greater than 10 (11.1) indicating that small amount of Ce is pre-inserted into the α-MnO_2_ structure.

**FIGURE 2:**
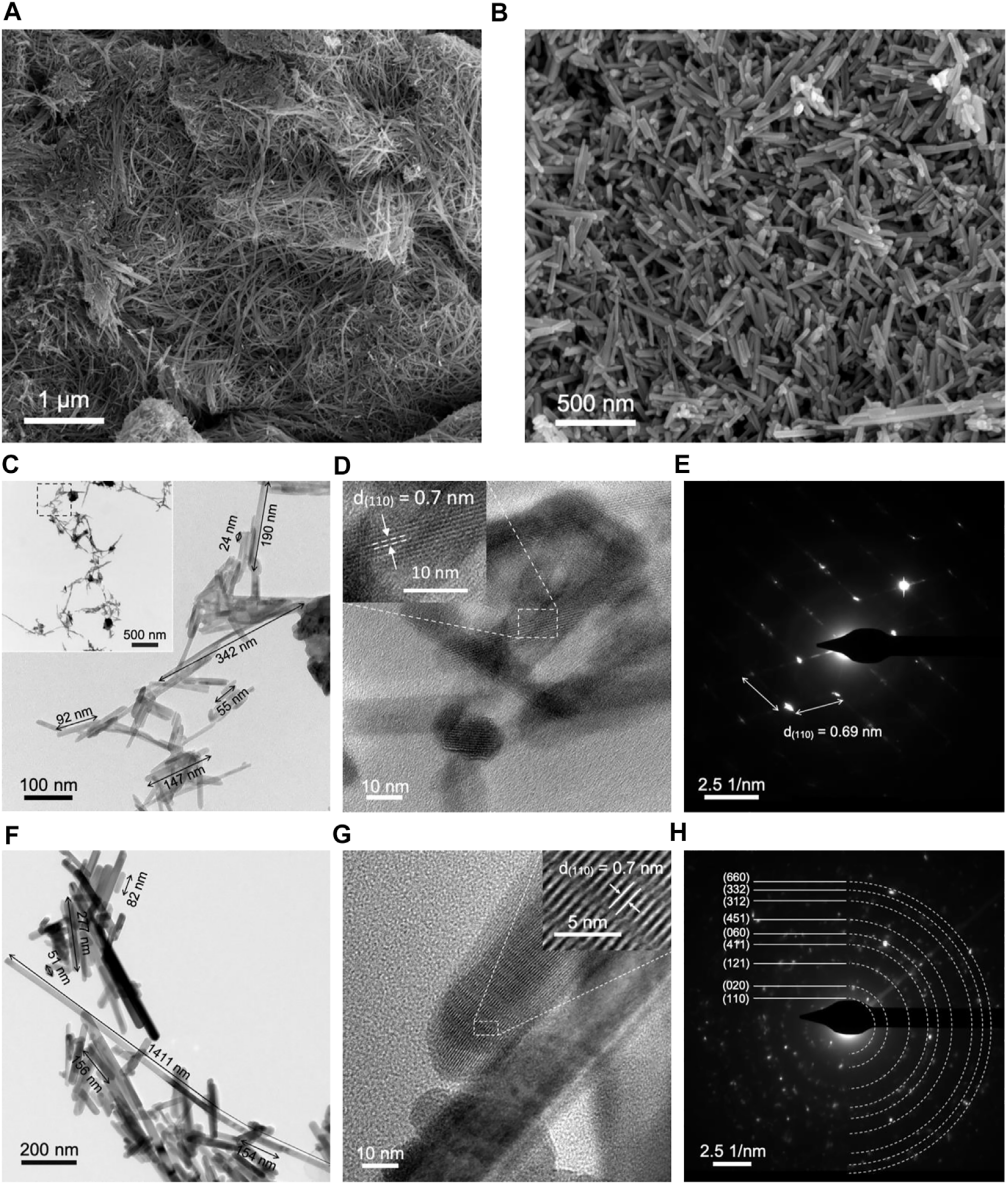
SEM images of **(A)** MnO_2_ nanofibers and **(B)** Ce-MnO_2_ nanorods. TEM, HRTEM and SAED pattern images of **(C–E)** MnO_2_ and **(F–H)** Ce-MnO_2_.

SEM analysis provides high-resolution imaging at different magnifications by scanning the surface of a solid sample with an electron beam. It is an analytical tool useful for observing the shape and the size of particles and the morphology of solid materials. Figures 2A, B present the SEM images at two different magnifications of MnO_2_ and Ce-MnO_2_, respectively. The micrographs display homogeneous morphologies for both MnO_2_ and Ce-MnO2, whilst different particle shapes and sizes were clearly observed. Figure 2A shows self-assembled nanofibers of MnO_2_ with diameters between 5 and 15 nm. However, well-defined nanorods (with average diameter and length of 50 and 500 nm, respectively) are perceived in Figure 2B for Ce-MnO_2_. It should be noted that several reports in literature mentioned that different MnO_2_ particle shapes (nanowires, urchin-like, nanorods, nanospheres, etc.) and sizes (from nm to μm) can be formed by hydrothermal method depending on several factors, including the reactants, pH conditions, temperature, and duration (Cheng et al., 2006; Duan et al., 2012; Su et al., 2013; Yin et al., 2015). It is then obvious that the presence of Ce^4+^ ions affected the morphology and the particle shape and size (from nanofibers to nanorods) of the product from the hydrothermal reaction.

Transmission electron microscopy (TEM) generates more sensitive images than SEM in the scale of sub-micrometers and nanometers. TEM imaging is used for morphological and structural characterization and dimensional measurements of nanostructures, atomic lattice spacing, and cell diameter with a resolution as low as a fraction of an Angstrom (1 Å = 10^–10^ m). TEM images of MnO_2_ and Ce-MnO_2_ are given in Figures 2C–H. The image and inset in Figures 2C, F confirm the morphologies and sizes observed in SEM analysis for MnO_2_ (nanofibers) and Ce-MnO_2_ (nanorods), respectively. The average length of MnO_2_ nanofibers is approximately 142 nm, whilst the average length of Ce-MnO_2_ nanorods is estimated to be 355 nm. High resolution TEM (HRTEM) imaging and selected area electron diffraction (SAED) patterns enable the direct measurement of lattice spacing between atomic planes in crystalline samples. Figures 2D, E present HRTEM images and SAED patterns of MnO_2_ crystals. The lattice spacing in MnO_2_ crystal estimated from the HRTEM images was 0.7 nm, corresponding to (110) crystal plane. Figures 2G, H present HRTEM images and SAED patterns of Ce-MnO_2_ crystals, which reveal the same lattice spacing as MnO_2_ sample. According to the SAED of Figures 2E, H, one shows an obvious two-dimensional lattice structure, and the other is a diffraction ring structure, which corresponds to monocrystalline and polycrystalline structures, for MnO_2_ and Ce-MnO_2_, respectively.

### 3.2 Electrochemical testing of MnO_2_-based cathodes

#### 3.2.1 Cyclic voltammetry

**FIGURE 3:**
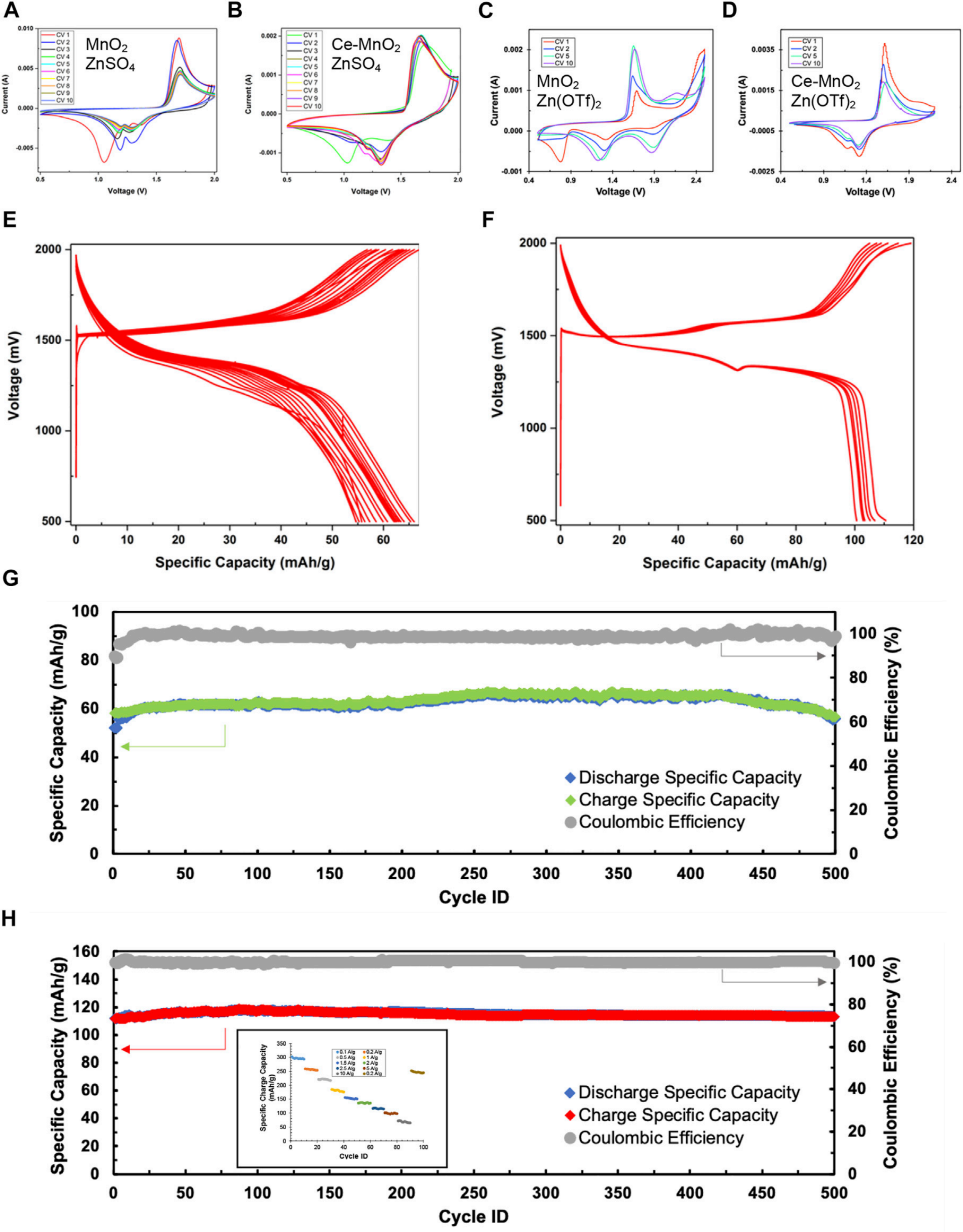
Cyclic voltammograms of **(A)** MnO_2_ and **(B)** Ce-MnO_2_ cathode materials with 3 M ZnSO_4_, and **(C)** MnO_2_ and **(D)** Ce-MnO_2_ with 3 M Zn(CF_3_SO_3_)_2_ tested in half-cell ARZIBs using Zn metal as reference and counter electrode at a scan rate of 1 mV/s. Voltage profiles during galvanostatic charge/discharge (GCD) at current density of 2500 mA/g with 3 M ZnSO_4_ of **(E)** MnO_2_ and **(F)** Ce-MnO_2_. Cycling stability measurements during GCD cycling of **(G)** MnO_2_ and **(H)** Ce-MnO_2_ cathode in 500 cycles (inset: rate capability).

Cyclic voltammetry (CV) is a widely used technique in electrochemistry. CV is a powerful tool to study the electrochemical behavior of a system by systematic study of current-voltage measurements of a given electrochemical cell. The oxidation or reduction taking place at the electrode surface is related with the potential of the electrode. The more negative potential implies more strongly reducing electrode and on the other hand more positive potential implies more strongly oxidizing electrode. Figures 3A, B presents the consecutive cyclic voltammograms of MnO_2_ and Ce-MnO_2_ used as working electrodes in half-cell ARZIBs using Zn metal as reference and counter electrode and 3 M ZnSO_4_ aqueous electrolyte. All peak positions identified for both half-cells are summarized in Table 1. Similar results were obtained in literature, which resembles the shape of CV curve in this study, as well as almost identical peak positions (Shi et al., 2020; Zang et al., 2020; Cai et al., 2021). In the return scan (reduction), two peaks appear at around 1.2 V and 1.3 V vs. Zn/Zn^2+^, which can be attributed to Zn^2+^ ions insertion into the MnO_2_ host structure wherein Mn^4+^ is reduced to lower valences (Figure 3A) (Cai et al., 2021). The first oxidation scan of MnO_2_ nanofibers cathode presents two peaks at approximately 1.6 V and 1.7 V vs. Zn/Zn^2+^, corresponding to the oxidation of Mn species back to Mn^4+^ and the de-insertion of Zn^2+^ ions. The only inconsistency lies in the cathodic region of the first CV scan, which showed a reduction peak positioned past 1.0 V, which also appeared for the Ce-MnO_2_ cell. In addition, the intensity of the peaks from both oxidation and reduction have been shown to decrease after CV2. On a positive note, consistent overlapping of the peaks from CV3 until CV10 was also observed, which signifies good reproducibility for long-term cycling. The CV of Ce-MnO_2_ in Figure 3B shows two peaks in the cathodic region at 1.2 and 1.3 V vs. Zn/Zn^2+^. Two consistent anodic peaks that are associated with the oxidation of Mn species back to Mn^4+^ were observed at 1.6 V (prominent) and 1.7 V (shoulder) vs. Zn/Zn^2+^. A peak at 1.1 V vs. Zn/Zn^2+^ was present in the first CV scan, which then shifted to around 1.2 V in the later scans. A slight increase in peak intensity was observed in the first two scans, which then remained consistent until CV 10. After CV 5, the peaks overlap which indicates favorable reproducibility upon further cycling. Generally, the number of peaks in the anodic and cathodic region are equivalent and not too far apart, indicating the reversible insertion/de-insertion of Zn^2+^ ions. Overall, both cells have shown good reproducibility and identical electrochemical peaks, indicating reversible insertion/de-insertion of Zn^2+^ ions. However, Ce-MnO_2_ half-cell has shown better reproducibility with almost identical peak positions and intensities throughout the ten cycles.

**TABLE 1:** Summary of the peak positions identified from cyclic voltammograms of MnO_2_ and Ce-MnO_2_ half-cells against Zn/Zn^2+^ in ZnSO_4_ and Zn(CF_3_SO_3_)_2_ electrolytes.

	ZnSO_4_		Zn(CF_3_SO_3_)_2_	
	Oxidation	Reduction	Oxidation	Reduction
MnO_2_	1.6 V	1.0 V (CV1)	1.7 V	0.8 V (CV1-2)
	1.7 V	1.2 V	2.1 V	1.3 V
		1.3 V		1.9 V
Ce- MnO_2_	1.6 V	1.1 V (CV1-2)	1.6 V	1.1 V (CV1-2)
	1.7 V	1.2 V	1.7 V	1.2 V
		1.3 V		1.3 V
		1.4 V (CV1)		

For comparison, CV was repeated for MnO_2_ and Ce-MnO_2_ coin cells using 3 M Zn(CF_3_SO_3_)_2_ as aqueous electrolyte (also known as Zn(OTf)_2_). Cyclic voltammograms for each MnO_2_ and Ce-MnO_2_ half-cell against Zn/Zn^2+^ are presented in Figures 3C, D. The peak positions are summarized in Table 1 against that of ZnSO_4_ electrolyte. The CV scan for MnO_2_ nanofiber material (Figure 3C) displays two cathodic peaks positioned at 1.7 and 2.1 V. The second peak at 2.1 V only appears to be present from CV5 and beyond. As for the reduction, three peaks appear at 0.8, 1.3, and 1.9 V, with an additional shoulder peak appearing at 2.1 V. However, the first peak located 0.8 V gradually decreased in intensity with each CV scan and completely disappeared from CV5 and beyond. The peak intensities have increased from CV1, reaching similar intensities in CV5 and CV10, as evident in the overlap. In terms of reproducibility, CV scan for MnO_2_ nanofiber material with ZnSO_4_ electrolyte in Figure 3A has shown higher reproducibility than that of Zn(CF_3_SO_3_)_2_ electrolyte in Figure 3C, which might be due to the secondary reactions of dissolution of Ti current collector in Zn(CF_3_SO_3_)_2_ at high voltages. Nevertheless, more distinct peaks were achieved when Zn(CF_3_SO_3_)_2_ electrolyte was used. The cyclic voltammogram of Ce-MnO_2_ half-cell (Figure 3D) presents peaks at similar positions seen in Figure 3B (anodic: 1.6 and 1.7 V; cathodic: 1.1, 1.2, and 1.3 V). The peak intensities appear to decrease with longer cycling and stabilize between CV5 and CV10. Compared to Figure 3B, this CV scan is more consistent with the peak positions, which indicates good recyclability.

#### 3.2.2 Cycling performance

To evaluate the electrochemical performance of α-MnO_2_ and Ce-inserted α-MnO_2_ as cathode materials for aqueous ZIBs, half-cells were prepared and cycled in battery analyzers. The charge rate (current density) in the GCD cycling of MnO_2_ and Ce-MnO_2_ was fixed to 2500 mA/g. Figures 3E, F shows voltage profiles (changes of voltage with specific capacity) during consecutive GCD cycles of MnO_2_ and Ce-MnO_2_ cathode materials tested in half-cell ARZIBs using Zn metal as reference and counter electrodes and 3 M ZnSO_4_ electrolyte. The first discharge and charge cycles of MnO_2_ cathode materials present reduction and oxidation plateaus at 1300 mV and 1500 mV vs. Zn/Zn^2+^, respectively (Figure 3E). For the subsequent GCDs, the same plateaus exist with a decrease in the specific capacity from one cycle to another. A good accordance between the CV results and the GCD plots was perceived. The GCD cycles of Ce-MnO2 present two plateaus in charge and two plateaus in discharge. The GCD cycles of Ce-MnO_2_ showed better stability (Figure 3F) than those of MnO_2_.

Figures 3G, H present the changes of specific charge and discharge capacities and Coulombic efficiency (CE) in 500 cycles. The first specific charge and discharge capacities of 55 and 52 mA h/g for MnO_2_, as shown in Figure 3G. The charge specific capacity undergoes a little increase and then stabilizes after 10 cycles, with CE reaching almost 100%. The specific charge capacity was retained at about 65 mA h/g for the rest of GCD cycles. Similar results were observed with Ce-MnO_2_ cathode materials (Figure 3H). It is markedly noted that Ce-MnO_2_ exhibited a more stable performance and much higher specific capacity values than MnO_2_, with a specific capacity of approximately 115 mA h/g throughout the 500 cycles. The pre-insertion of Ce^4+^ ions into the tunnels of α-MnO_2_ improves the specific capacity of the battery. The average specific charge capacities of Ce-MnO_2_ after 10 GCD cycles at each of the following rates 100, 200, 500, 1000, 1500, 2000, 2500, 5000, 10000 were 297, 256, 222, 183, 153, 136, 116, 97, and 68 mA h/g, respectively. The Ce-MnO_2_ retained an average specific charge capacity of 246 mA h/g at 200 mA/g indicating a robust chemical structure and excellent rate capability.

### 3.3 Post-mortem analysis

Characterization techniques, particularly SEM/EDX and TEM, were used to observe the changes in the cathode material before and after cycling. Before cycling, the cathode only consists of the casted slurry components (active material, binder, carbon black). During discharge, Zn^2+^ ions are released from the anode and travel through the electrolyte and inserted within the cathode material. The Zn^2+^ ions undergo de-insertion from the cathode and travel back to the anode during charge in secondary batteries. The following results from post-mortem analysis revealed the rechargeable nature of the half-cells.

**FIGURE 4:**
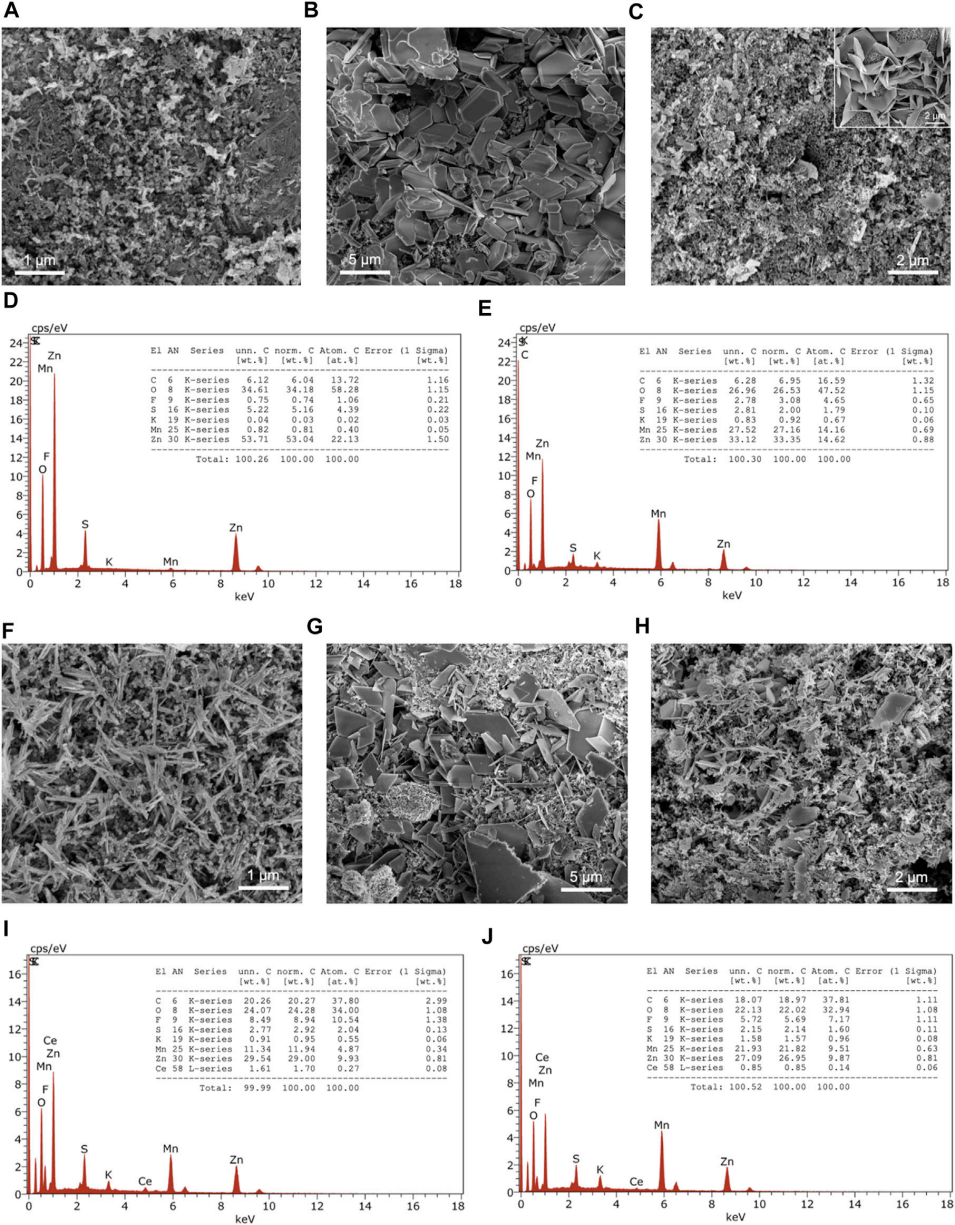
SEM images before cycling, after discharge, and after charge of **(A–C)** MnO_2_ with EDX spectra **(D)** after discharge and **(E)** after charge, and **(F–H)** Ce-MnO_2_ cathodes with EDX spectra **(I)** after discharge and **(J)** after charge. Inset of SEM image of microsheets.

SEM imaging of the cathode’s surface before cycling shows MnO_2_ microfibers mixed with the slurry components (Figure 4A). However, the components are not evenly distributed in the slurry, which may be the reason for the low specific capacities observed in GCD. Upon discharging, the appearance of the cathode has drastically changed from microfibers to smooth, three-dimensional units of varying thicknesses due to stacking (Figure 4B). Finally, the charged state of the MnO_2_ cathode reverted back to its original appearance, with the exception of microsheets protruding out of the cathode in certain areas (Figure 4C). The same observation was found in a birnessite-type MnO_2_ in discharged states (Wang et al., 2019). The growth of the microsheets were associated with the formation of zinc sulfate hydroxide hydrate, [Zn(OH_2_)_3_ZnSO_4_⋅*x*H_2_O]. The Ce-inserted MnO_2_ cathode consists of nanorods, which are evident the micrograph before cycling (Figure 4F). After discharging to 0.5 V, plate-like structures are observed on the surface, indicating a phase transformation (Figure 4G). In the charged state, the cathode material has almost completely reverted back to the pristine state, with some of the plate-like units remaining on the surface (Figure 4H).

EDX analysis of MnO_2_ cathode revealed a large Zn/Mn ratio, which indicates the insertion of Zn^2+^ in the MnO_2_ structure (zincation) and the formation of other Zn-based compounds (Figure 4D). In the charged state, the Zn/Mn ratio decreased to 1 (Figure 4E). Similar observations were seen with Ce-MnO_2_ cathode. Upon discharging, the Zn/Mn ratio was 2, which indicates that two Zn^2+^ ions were inserted in the cathode host structure (full zincation) (Figure 4I). After charging, the Zn/Mn ratio also decreased to 1 (Figure 4J). This may suggest that the de-insertion of Zn^2+^ ions was incomplete or other Zn compounds have formed, as shown in the formation of microsheets from SEM analysis (Figure 4C).

#### 3.3.1 TEM of cathode materials

**FIGURE 5:**
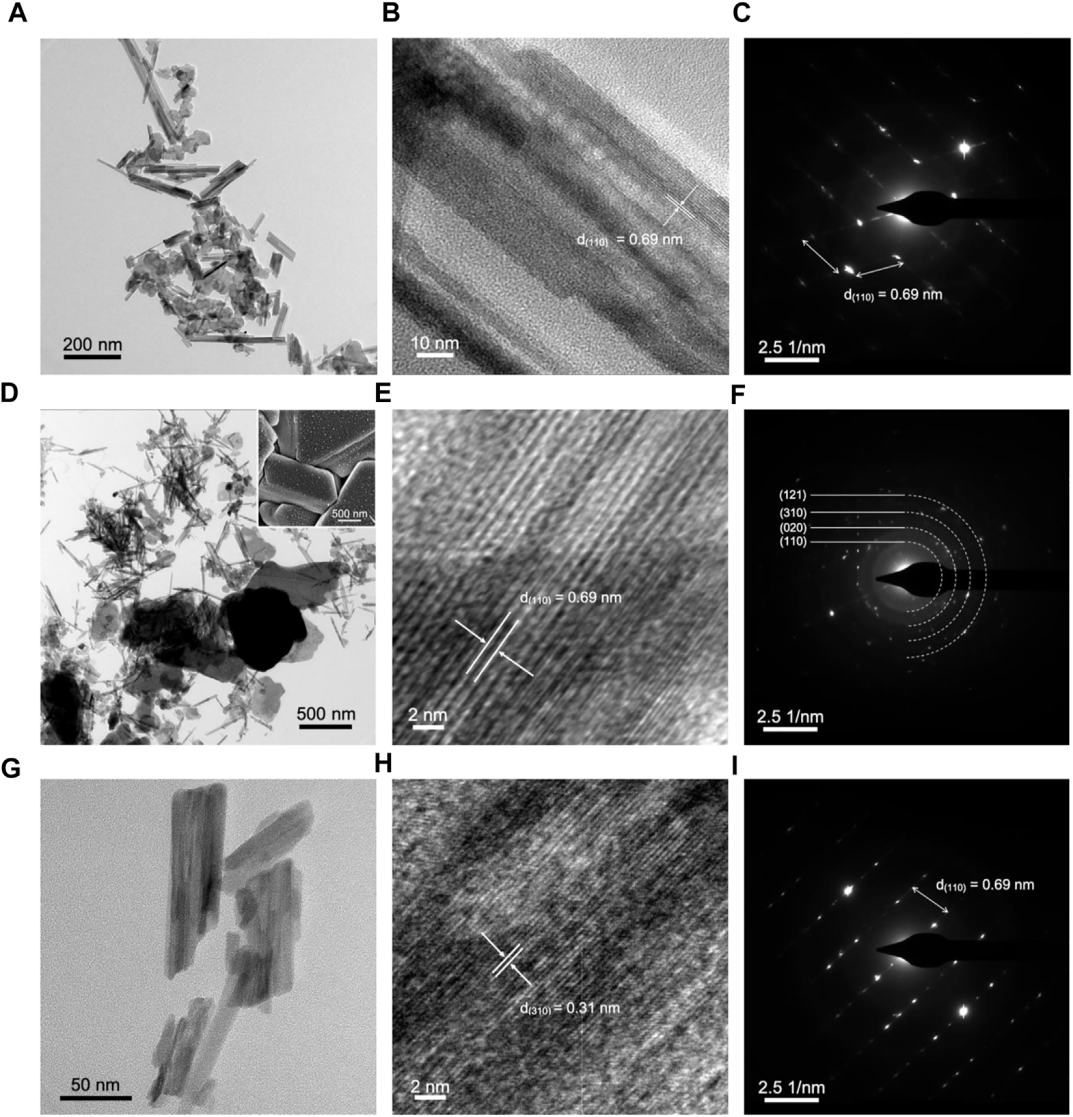
TEM, HRTEM, and SAED pattern images of MnO_2_ cathode **(A–C)** before cycling, **(D–F)** after discharge, and **(G–I)** after charge. Inset of SEM image of cathode after discharge.

TEM analysis of the MnO_2_ cathode supports the images produced from SEM, which revealed a nanofiber structure (Figure 5A). The crystal plane that was evident in all stages is (110), which has a d-spacing of 0.69 nm in α-MnO_2_·0.15 H_2_O. In Figure 5D, the structures formed during discharge in SEM imaging was also observed in TEM, with lengths of over 1 µm. Furthermore, a change from monocrystalline to polycrystalline phase was evident in the SAED pattern images before cycling (Figure 5C) and after discharge (Figure 5F). Besides (110) crystal plane, three other planes were observed including (020), (310), and (121), which are the main crystal planes of α-MnO_2_·0.15 H_2_O. After charge, a return to monocrystalline phase was observed, which revealed crystal plane (110). This indicates that the anode material is capable of reverting back to the original state before cycling, which is equivalent to good reversibility, as reflected in electrochemical findings.

**FIGURE 6:**
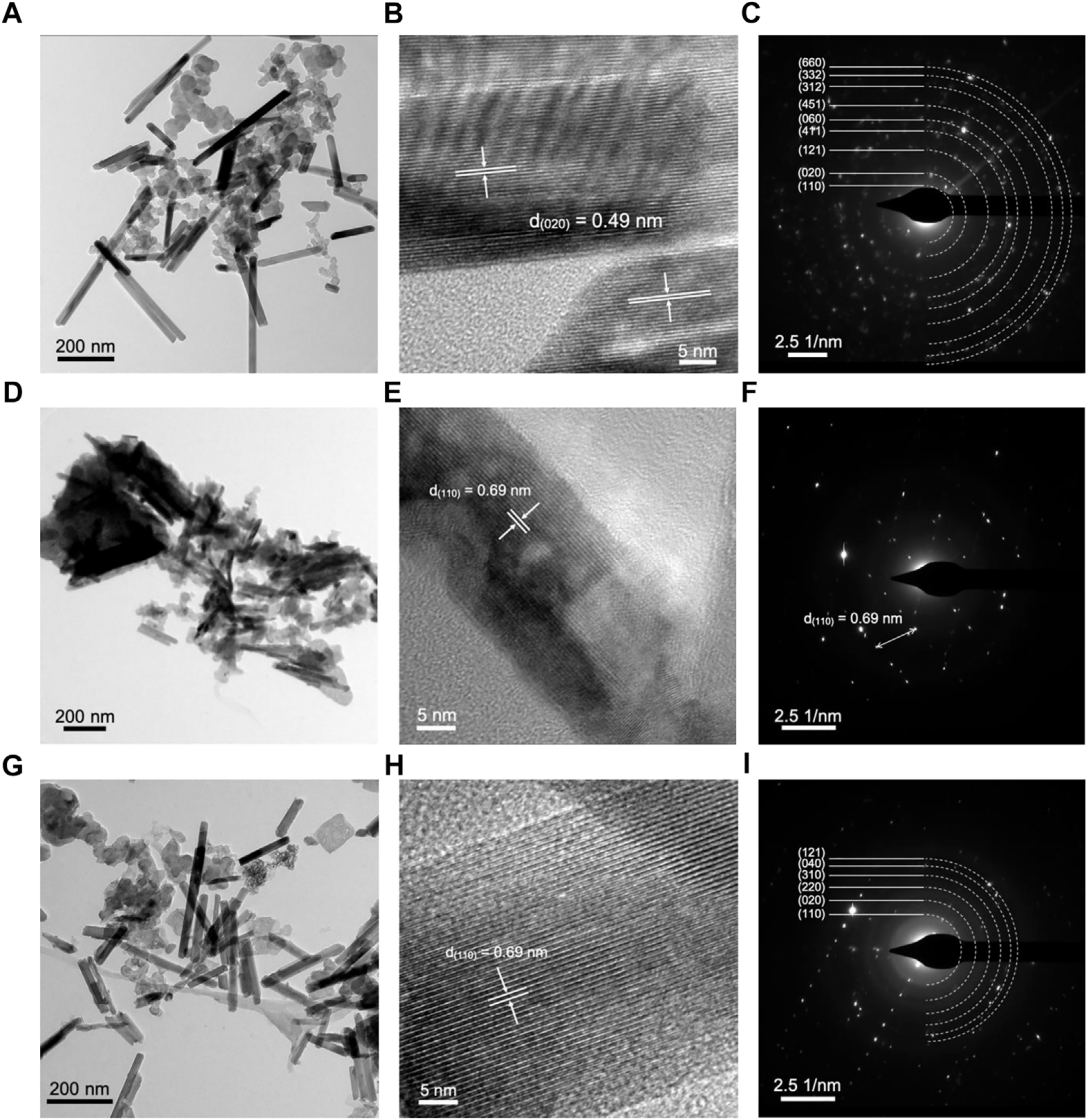
TEM, HRTEM, and SAED pattern images of Ce‐MnO2 cathode **(A–C)** before cycling, **(D–F)** after discharge, and **(G–I)** after charge.

The TEM imaging of Ce-MnO_2_ cathode in post-mortem analysis showed the nanorod structures present in SEM analysis (Figure 6A). Similar to MnO_2_, Ce-inserted

MnO_2_ had d-spacing that matched well with that of α-MnO_2_·0.15 H_2_O, especially the characteristic (110) crystal plane. Before cycling, Ce-MnO_2_ cathode was polycrystalline, as shown in the SAED pattern image in Figure 6C. After discharge, the phase changed to monocrystalline. The phase returned back to polycrystalline after charge, indicating good reversibility of the cathode.

Overall, post-mortem analysis has revealed that the cathode is capable of reverting back to the original structure, which is indicative of good reversibility. For both cathodes, the formation of microstructures were evident in SEM and TEM analysis in the discharged state. In particular, MnO_2_ cathode showed a more significant change from nanofibers to stacked 3-D units. However, these structures were replaced with the original nanofibers and scattered microsheets, which were previously reported to be [Zn(OH_2_)_3_ZnSO_4_⋅*x*H_2_O]. In all states of the cathode, the (110) crystal plane was observed in HRTEM and SAED pattern images. Therefore, the original structure of MnO_2_ was preserved in both pristine and Ce-inserted cathodes.

## 4 Conclusion

In this work, hydrothermal method was used to synthesize MnO_2_ and Ce-MnO_2_ nanostructures. XRD analysis confirmed the formation of α-MnO_2_ structures: α-MnO_2_·0.15 H_2_O and Ce-inserted α-MnO_2_·0.15 H_2_O. Raman spectroscopy confirmed the formation of tunnel-type α-MnO_2_ structures with the presence of Mn–O–Mn bonds in MnO_6_ octahedra and terminal Mn–O bonds stretching vibrations. SEM imaging revealed the formation of nanomaterials with different morphologies: nanofibers for MnO_2_ and nanorods for Ce-MnO_2_. The electrochemical testing by CV and GCD demonstrate that MnO_2_ nanofibers as ARZIB cathode can deliver a reversible specific capacity of 65 mA h/g throughout the majority of cycles. Meanwhile, Ce-MnO_2_ nanorods cathode exhibited better performance than MnO_2_ with a specific capacity of 115 mA h/g with high capacity retention over 500 GCD cycles. These results indicate that the pre-insertion of Ce^4+^ ions into the tunnels of MnO_2_ structure enhances the performance of Zn/MnO_2_ battery. Post-mortem analysis revealed that the cathode retained the structure of α-MnO_2_·0.15 H_2_O for the most part. Significant structural changes were observed for the MnO_2_ cathode after discharge due to zincation. Good reversibility was indicated in the results from post-mortem analysis, which supports the electrochemical findings. Further enhancements can be done in the future by searching for more stable electrolytes and nanostructured layered structures.

## Data Availability

The original contributions presented in the study are included in the article/supplementary material, further inquiries can be directed to the corresponding author.

## References

[B1] Alfaruqi M. H. Gim J. Kim S. Song J. Jo J. Kim S. (2015). Enhanced reversible divalent zinc storage in a structurally stable α-MnO2 nanorod electrode. J. Power Sources 288, 320–327. 10.1016/j.jpowsour.2015.04.140

[B2] Andre D. Hain H. Lamp P. Maglia F. Stiaszny B. (2017). Future high-energy density anode materials from an automotive application perspective. J. Mater Chem. A Mater 5, 17174–17198. 10.1039/C7TA03108D

[B3] Cai X. Li H. Li J. Yan H. Liu Y. Yu H. (2021). Hydrothermal synthesis of β-MnO2 nanorods for highly efficient zinc-ion storage. Ionics (Kiel). 27, 3943–3950. 10.1007/s11581-021-04188-6

[B4] Cheng F. Zhao J. Song W. Li C. Ma H. Chen J. (2006). Facile controlled synthesis of MnO2 nanostructures of novel shapes and their application in batteries. Inorg. Chem. 45, 2038–2044. 10.1021/ic051715b 16499364

[B5] Costa C. M. Barbosa J. C. Gonçalves R. Castro H. del Campo F. J. Lanceros-Méndez S. (2021). Recycling and environmental issues of lithium-ion batteries: Advances, challenges and opportunities. Energy Storage Mater 37, 433–465. 10.1016/j.ensm.2021.02.032

[B6] Duan X. Yang J. Gao H. Ma J. Jiao L. Zheng W. (2012). Controllable hydrothermal synthesis of manganese dioxide nanostructures: Shape evolution, growth mechanism and electrochemical properties. CrystEngComm 14, 4196. 10.1039/c2ce06587h

[B7] Fang G. Zhou J. Pan A. Liang S. (2018). Recent advances in aqueous zinc-ion batteries. ACS Energy Lett. 3, 2480–2501. 10.1021/acsenergylett.8b01426

[B8] Hsu Y. K. Chen Y. C. Lin Y. G. Chen L. C. Chen K. H. (2011). Reversible phase transformation of MnO2 nanosheets in an electrochemical capacitor investigated by *in situ* Raman spectroscopy. Chem. Commun. 47, 1252–1254. 10.1039/c0cc03902k 21103502

[B9] Huang J. Q. Lin X. Tan H. Du X. Zhang B. (2020). Realizing high-performance Zn-ion batteries by a reduced graphene oxide block layer at room and low temperatures. J. Energy Chem. 43, 1–7. 10.1016/j.jechem.2019.07.011

[B10] Ji C. Ren H. Yang S. (2015). Control of manganese dioxide crystallographic structure in the redox reaction between graphene and permanganate ions and their electrochemical performance. RSC Adv. 5, 21978–21987. 10.1039/c5ra01455g

[B11] Julien C. Massot M. Baddour-Hadjean R. Franger S. Bach S. Pereira-Ramos J. P. (2003). Raman spectra of birnessite manganese dioxides. Solid State Ion. 159, 345–356. 10.1016/S0167-2738(03)00035-3

[B12] Kundu D. Adams B. D. Duffort V. Vajargah S. H. Nazar L. F. (2016). A high-capacity and long-life aqueous rechargeable zinc battery using a metal oxide intercalation cathode. Nat. Energy 1, 16119. 10.1038/nenergy.2016.119

[B13] Li Y. Dai H. (2014). Recent advances in Zinc-air batteries. Chem. Soc. Rev. 43, 5257–5275. 10.1039/c4cs00015c 24926965

[B14] Liu B. Sun Y. Liu L. Xu S. Yan X. (2018). Advances in manganese-based oxides cathodic electrocatalysts for Li–air batteries. Adv. Funct. Mater 28, 1704973. 10.1002/adfm.201704973

[B15] Liu C. Chi X. Huang J. Liu Y. (2021). A high-voltage rechargeable alkaline Zn–MnO4− battery with enhanced stability achieved by highly reversible MnO4−/MnO42− redox pair. Mater Today Energy 20, 100680. 10.1016/j.mtener.2021.100680

[B16] Ming J. Guo J. Xia C. Wang W. Alshareef H. N. (2019). Zinc-ion batteries: Materials, mechanisms, and applications. Mater. Sci. Eng. R Rep. 135, 58–84. 10.1016/j.mser.2018.10.002

[B17] Post J. E. (1999). Manganese oxide minerals: Crystal structures and economic and environmental significance. Proc. Natl. Acad. Sci. U. S. A. 96, 3447–3454. 10.1073/pnas.96.7.3447 10097056 PMC34287

[B18] Shahjalal M. Shams T. Islam Md.E. Alam W. Modak M. bin Hossain S. (2021). A review of thermal management for Li-ion batteries: Prospects, challenges, and issues. J. Energy Storage 39, 102518. 10.1016/j.est.2021.102518

[B19] Shi M. Xiao P. Yang C. Sheng Y. Wang B. Jiang J. (2020). Scalable gas-phase synthesis of 3D microflowers confining MnO2 nanowires for highly-durable aqueous zinc-ion batteries. J. Power Sources 463, 228209. 10.1016/j.jpowsour.2020.228209

[B20] Su D. Ahn H. J. Wang G. (2013). Hydrothermal synthesis of α-MnO2 and β-MnO 2 nanorods as high capacity cathode materials for sodium ion batteries. J. Mater Chem. A Mater 1, 4845. 10.1039/c3ta00031a

[B21] Tang B. Shan L. Liang S. Zhou J. (2019). Issues and opportunities facing aqueous zinc-ion batteries. Energy Environ. Sci. 12, 3288–3304. 10.1039/c9ee02526j

[B22] Wang J. Wang J.-G. Liu H. Wei C. Kang F. (2019). Zinc ion stabilized MnO2 nanospheres for high capacity and long lifespan aqueous zinc-ion batteries. J. Mater Chem. A Mater 7, 13727–13735. 10.1039/C9TA03541A

[B23] Wu T. H. Zhang Y. Althouse Z. D. Liu N. (2019). Nanoscale design of zinc anodes for high-energy aqueous rechargeable batteries. Mater Today Nano 6, 100032. 10.1016/j.mtnano.2019.100032

[B24] Xu C. Chen Y. Shi S. Li J. Kang F. Su D. (2015). Secondary batteries with multivalent ions for energy storage. Sci. Rep. 5, 14120. 10.1038/srep14120 26365600 PMC4568479

[B25] Xu C. Li B. Du H. Kang F. (2012). Energetic zinc ion chemistry: The rechargeable zinc ion battery. Angewandte Chemie - International Edition. 10.1002/anie.201106307 22170816

[B26] Xu W. Wang Y. (2019). Recent progress on zinc-ion rechargeable batteries. Nanomicro Lett. 11, 90. 10.1007/s40820-019-0322-9 34138036 PMC7770952

[B27] Yan J. Wang J. Liu H. Bakenov Z. Gosselink D. Chen P. (2012). Rechargeable hybrid aqueous batteries. J. Power Sources 216, 222–226. 10.1016/j.jpowsour.2012.05.063

[B28] Yin B. Zhang S. Jiang H. Qu F. Wu X. (2015). Phase-controlled synthesis of polymorphic MnO2 structures for electrochemical energy storage. J. Mater Chem. A Mater 3, 5722–5729. 10.1039/c4ta06943a

[B29] Zang X. Li L. Sun Z. Boukherroub R. Meng J. Cai K. (2020). A simple physical mixing method for MnO2/MnO nanocomposites with superior Zn2+ storage performance. Trans. Nonferrous Metals Soc. China 30, 3347–3355. 10.1016/S1003-6326(20)65466-8

[B30] Zhang N. Cheng F. Liu J. Wang L. Long X. Liu X. (2017). Rechargeable aqueous zinc-manganese dioxide batteries with high energy and power densities. Nat. Commun. 8, 405. 10.1038/s41467-017-00467-x 28864823 PMC5581336

[B31] Zhou S. Wu X. Du H. He Z. Wu X. Wu X. (2022). Dual metal ions and water molecular pre-intercalated δ-MnO2 spherical microflowers for aqueous zinc ion batteries. J. Colloid Interface Sci. 623, 456–466. 10.1016/j.jcis.2022.05.018 35597015

[B32] Zhu K. Wu T. Sun S. Wen Y. Huang K. (2020). Electrode materials for practical rechargeable aqueous Zn-ion batteries: Challenges and opportunities. ChemElectroChem 13, 2714–2734. 10.1002/celc.202000472

